# Utilizing the Delphi Method to Develop Undergraduate Medical Education Learning Objectives to Address Medical Care of Gender and Sexually Diverse Individuals

**DOI:** 10.7759/cureus.70779

**Published:** 2024-10-03

**Authors:** Raymond Reynolds, Jacob Knight, Bridget Dorsey, Caitlin Phillips, Vismaya Kharkar, Kayla Blickensderfer, Cornelia Keyser, Brendan Hatch, Erin Connelly, Julia Decker, Lisa Diamond, Michael Battistone, Andrea Barker, Julie Thomas

**Affiliations:** 1 Internal Medicine, University of Utah, Salt Lake City, USA; 2 Psychology, University of Utah, Salt Lake City, USA; 3 Internal Medicine, Veterans Affairs Medical Center, Salt Lake City, USA; 4 Rheumatology, University of Utah, Salt Lake City, USA

**Keywords:** diversity, diversity and inclusion, gender, lgbtq, lgbtq medicine, medical education, sexuality

## Abstract

Introduction

Gender and sexually diverse (GSD) individuals disproportionately experience worse outcomes, bias, discrimination, and inequities in their care. Many avoid seeking healthcare due to fear of discrimination and mistreatment. One method for improvement focuses on specific GSD medical care training for undergraduate medical education (UME) learners. Efforts to standardize GSD care in UME are present, as displayed by the competencies put forth by the Association of American Medical Colleges (AAMC); however, these attempts resulted in broad themes that can be challenging to implement. The need for specific and easily implementable learning objectives exists.

Methods

We aimed to create a set of learning objectives specific to GSD care by utilizing the Delphi Method to develop consensus. Three hundred and seventy-nine individuals were invited to participate in this study, which involved four iterative rounds of expert participation. In round one, the expert panel received an initial questionnaire comprising published learning competencies and items. The panel was requested to review and propose additional items. The research team then consolidated and structured these items into learning objectives. In round two, the expert panel was asked to review these objectives and edit language to reflect appropriate, inclusive language. In round three, the expert panel was asked to rate the importance of each learning objective using a 5-point Likert scale (1 =not at all important; 5 =extremely important). In round four, experts were given the overall panel’s mean and mode rating for each objective, reminded of their rating, and asked to make a final rating. Learning objectives rated 4 or 5 (“very important” or “extremely important”) by at least 80% of experts were determined to be at consensus. The researchers then further examined objectives that had 100% respondent rating of either 4 or 5, thus achieving universal consensus by our expert panel.

Results

Although 59 individuals agreed to participate in the study, 31 individuals engaged in at least one round of the iterative process as part of the expert panel. The initial questionnaire comprised 30 competencies published by the AAMC and 32 published overlapping learning items. After round two, 79 learning objectives were created. This process eliminated 28 objectives, resulting in 51 succinct objectives that used inclusive and patient-centered language.

Conclusion

These learning objectives can easily be integrated into existing curricular structures in UME. They can be utilized to improve curricular education for future health professionals, with the final goal of improving health equity for GSD individuals.

## Introduction

Gender and sexually diverse (GSD) individuals experience bias, discrimination, and inequalities in health care [1,2]. Many GSD patients believe that healthcare professionals are inadequately trained to treat GSD individuals [2]. Concurrently, medical students feel that they do not receive sufficient curricular preparation to treat GSD patients and address their health inequities [3]. Educating future healthcare professionals about the specific needs of GSD individuals is essential in mitigating discrimination and improving the delivery of patient-centered and GSD-inclusive care [4-7]. Of note, the term GSD is used instead of terms such as lesbian, gay, bisexual, transgender, queer, intersex (LGBTQI), or sexual and gender minority (SGM) to emphasize the fluidity and complexity of sexuality and gender as part of the human condition rather than emphasizing exclusivity, hierarchy, or identity alone [1].

GSD individuals disproportionately experience worse health outcomes, including higher rates of mental health disorders, substance abuse, sexually transmitted infections, and cancers [1,8-10]. These patients often avoid seeking medical care because of discrimination, especially in healthcare environments, leading to delays in care [11]. Strikingly, about fifty percent of GSD individuals identifying as lesbian, gay, and bisexual, and nearly ninety percent of GSD individuals identifying as transgender or non-binary believed that healthcare professionals lacked appropriate knowledge and sensitivity to treat GSD individuals [2].

Medical students agree with this sentiment and want their current curriculum to deliver adequate knowledge about care for GSD persons [3]. In surveying medical school curriculum for GSD training, a 2011 Journal of the American Medical Association study of 176 allopathic and osteopathic medical schools in Canada and the United States reported that the median time dedicated to teaching GSD-related content in the entire curriculum was five hours (interquartile range of three to eight hours and total range of 0 to 32 hours); content varied wildly from one institution to the next [12]. In a 2015 study of 176 medical schools in the United States and Canada, 67% of medical students evaluated their GSD-related curriculum as fair or worse [13].

There are efforts to standardize GSD health throughout undergraduate medical education (UME). In 2014, the Association of American Medical Colleges (AAMC) published a set of 30 competencies to address GSD health inequalities to be incorporated into UME [4]. However, the AAMC competencies have been criticized as being too broad and vague, making it challenging to incorporate them into UME. In 2018, the Georgetown School of Medicine (GSOM) published a study incorporating a set of learning items written by the Vanderbilt School of Medicine (VSOM) with associated AAMC competencies [5]. While the VSOM learning items are concise, they were designed to be institution-specific for VSOM and address this institution's particular needs [5]. More recently, in 2022, Harvard Medical School (HMS) published a landmark study referencing the AAMC competencies and exploring how these can be utilized in undergraduate medical education curriculum development [14]. 

In addition to a curricular roadmap, a more specific and comprehensive list of GSD learning objectives using accurate language and reflecting the GSD experiences is needed for medical schools to effectively and efficiently incorporate GSD health-related content into UME. These learning objectives should be clear and organized so that any institution could incorporate them, including medical schools without adequate resources or faculty expertise. We aim to create a list of implementable learning objectives to teach GSD-related topics to UME learners to provide improved care for GSD individuals and potentially improve health equity.

This article was previously presented as a meeting abstract for the Western Group of Educational Affairs of the Association of American Medical Colleges in April 2023.

## Materials and methods

To create learning objectives, we chose to utilize the Delphi Method as our study framework, as this achieved consensus in an iterative process with a diverse group of professionals [15]. The consensus opinion is valuable when evidence is limited and is considered particularly relevant in medical education as it “extracts the profession’s collective knowledge” [16]. The Delphi method is an approach to developing consensus and involves the following ordered steps: (1) problem identification (2) literature review and development of an initial questionnaire (3) expert panel selection (4) poll experts iteratively (5) identify convergence of opinion (6) report the results [15].

**Figure 1:**
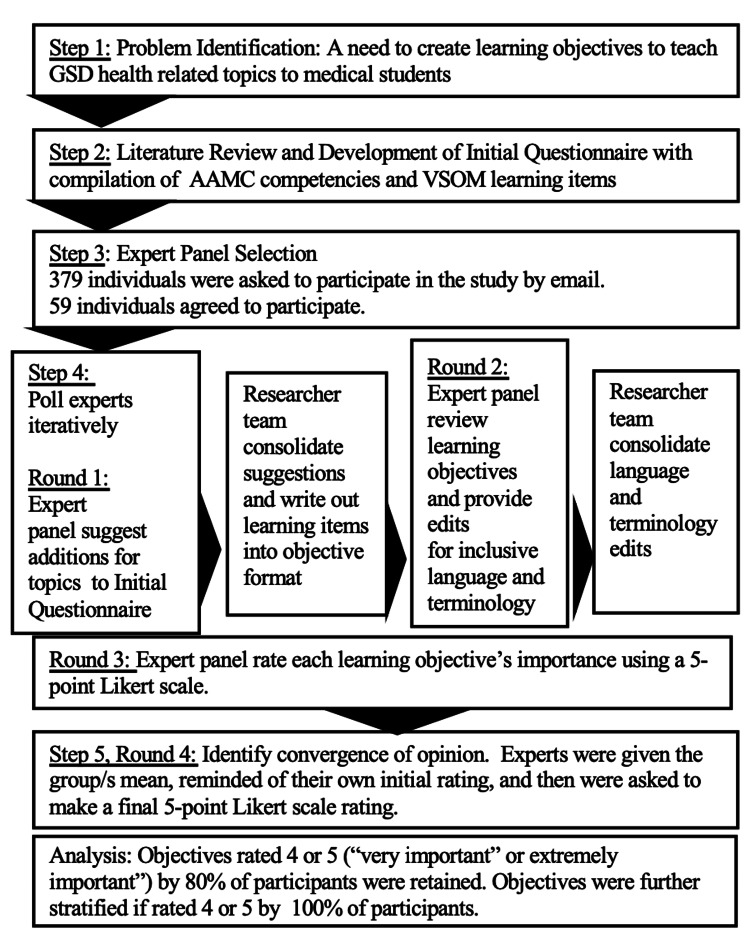
Flow model of the Delphi process used to assess and further develop learning objectives.

The specific methodology of this Delphi study can be seen in Figure 1.

Step one: problem identification

We identified a need to create implementable learning objectives devoted to GSD medical content to help teach UME learners.

Step two: literature review and development of the initial questionnaire

In developing a comprehensive list of topics to be included in GSD curricula, we queried articles related to improving UME inclusivity of GSD health topics up to September of 2021 using the following search terms: "education, medical, clinical, undergraduate” (and variations), "sexual and gender minorities” (and variations), and “lesbian, gay, bisexual, homosexual, queer or LGBTQI” (and variations). We focused on results with published competencies or learning objectives only. The search identified the following resources: DeVita et al. with associated VSOM GSD learning items and the 2014 AAMC implementing curricular and institutional climate changes to improve health care for individuals who are LGBTQI, gender nonconforming, or born with disorders of sexual development (DSD) [4,5]. These resources align most with the authors’ goal: to create a comprehensive list of GSD learning objectives that are easily integrated, accurate, and inclusive. Namely, the 2014 AAMC GSD competencies provided a validated, comprehensive list of content related to GSD health, and DeVita et al. provided easily integrated and concise VSOM learning items matched with the 2014 AAMC LGBTQI competencies [4,5]. We decided to use this combination as the initial questionnaire for our Delphi study (Table 1).

**Table 1:** Initial questionnaire This is the initial questionnaire used in our Delphi study and it is a compilation of the AAMC competencies and Vanderbilt School of Medicine learning objectives [4,5]. ID: infectious disease, STI: sexually transmitted infections, MSM: men who have sex with men, WSW: women having sex with women, HPV: human papillomavirus, LGBTQI: lesbian, gay, bisexual, transgender, queer, intersex.

Competencies from the AAMC (listed by domain)	Associated Learning Objectives
Competency Domain: Patient Care Gather essential and accurate information about patients and their conditions through history taking physical examination, and the use of laboratory data, imaging, and other tests by:	
Sensitively and effectively eliciting relevant information about sex anatomy, sex development, sexual behavior, sexual history, sexual orientation, sexual identity, and gender identity from all patients in a developmentally appropriate manner.	Communication / Interview Skills; Standardized Patient Cases; Embryology - Disorders of Sex Development (What are they? What are the current thoughts on treatment options? What are gender assignments?); Infectious Disease (ID) - sexually transmitted infections (STIs) in lesbians (What do/can they get?); ID - STI recommendations in men who have sex with men (MSM); HIV in MSM Anal Pap smears MSMs and need of Hepatitis A/HPV shot; Gay teen issues (psychological/sexual/coming out/identity development/schooling); Gender dysphoria versus Transgender; LGBTQI Teen Issues
Performing a complete and accurate physical exam with sensitivity to issues specific to the individuals described above at stages across the lifespan. This includes knowing when particulars of the exam are essential and when they may be unnecessarily traumatizing (as may be the case, for example, with repeated genital exams by multiple providers).	Standardized Patient Cases; Embryology - Disorders of Sex Development (What are they? What are the current thoughts on treatment options? What are gender assignments?); Exclusive women having sex with women (WSWs): Pap smear, Breast Exams, and HPV Screening; Anal Pap smear; MSMs and need of Hepatitis A/HPV shot; Gay teen issues (psychological/sexual/coming out/identity development/schooling); LGBTQI Teen Issues
Make informed decisions about diagnostic and therapeutic interventions based on patient information and preferences, up-to-date scientific evidence, and clinical judgment by:	
Describing the special health care needs and available options for quality care for transgender patients and for patients born with disorders of sexual development (DSD) (e.g., specialist counseling, pubertal suppression, elective and nonelective hormone therapies, elective and nonelective surgeries, etc.).	Embryology - Disorders of Sex Development (What are they? What are the current thoughts on treatment options? What are gender assignments?); Hormone Therapy Pharmacology; Transitioning options and associated risks; Puberty suppression in management of trans youth; Gender dysphoria versus Transgender
Counsel and educate patients and their families to empower them to participate in their care and enable shared decision-making by:	
Assessing unique needs and tailoring the physical exam and counseling and treatment recommendations to any of the individuals described above, taking into account any special needs, impairments, or disabilities	Standardized Patient Cases; ID - STIs in lesbians (What do/can they get?); ID - Vaginitis spread in lesbians; ID - STI recommendations in MSM; HIV in MSM; Availability/Efficacy of Rectal Microbicides; Exclusive women who have sex with women (WSW): Pap smear, Breast Exams, and HPV Screening; Anal Pap smear; MSMs and need of Hepatitis A/HPV shot; Lesbian Obesity; Increased heart disease rate in lesbians; Anal Cancer Risks, Treatments, Anal Pap in MSM; Lesbian nulliparity and risk of breast/ovarian/cervical cancer; Gay teen issues (psychological/sexual/coming out/identity development/schooling); Depression and Suicide Rates in LGBTQI teens/adults; Eating disorders in MSM; LGBTQI Teen Issues
Recognizing the unique health risks and challenges often encountered by the individuals described above, as well as their resources, and tailoring health messages and counseling efforts to boost resilience and reduce high-risk behaviors.	Substance Abuse Screening; Standardized Patient Cases; ID - STIs in lesbians (What do/can they get?); ID - Vaginitis spread in lesbians; ID - STI recommendations in MSM; HIV in MSM; Availability/Efficacy of Rectal Microbicides; Hormone Therapy Pharmacology; Lesbian Obesity; Increased heart disease rate in lesbians; Anal Cancer Risks, Treatments, Anal Pap in MSM; Lesbian nulliparity and risk of breast/ovarian/cervical cancer
Provide health care services to patients, families, and communities aimed at preventing health problems or maintaining health by:	
Providing effective primary care and anticipatory guidance by utilizing screening tests, preventive interventions, and health care maintenance for the populations described above (e.g., screening all individuals for inter-partner violence and abuse; assessing suicide risk in all youth who are gender nonconforming and/or identify as gay, lesbian, bisexual and/or transgender; and conducting screenings for transgender patients as appropriate to each patient’s anatomical, physiological, and behavioral histories).	Communication / Interview Skills; Depression Screening; Substance Abuse Screening; Embryology - Disorders of Sex Development (What are they? What are the current thoughts on treatment options? What are gender assignments?); ID - STIs in lesbians (What do/can they get?); ID - Vaginitis spread in lesbians; ID - STI recommendations in MSM; HIV in MSM; Availability/Efficacy of Rectal Microbicides; Exclusive WSWs: Pap smear, Breast Exams, and HPV Screening; Anal Pap smear; MSMs and need of Hepatitis A/HPV shot; Hormone Therapy Pharmacology; Gay teen issues (psychological/sexual/coming out/identity development/schooling); LGBTQI Teen Issues
Competency Domain: Knowledge for Practice	
Apply established and emerging biophysical scientific principles fundamental to health care for patients and populations by:	
Defining and describing the differences among sex and gender; gender expression and gender identity; gender discordance, gender nonconformity, and gender dysphoria; and sexual orientation, sexual identity, and sexual behavior.	Intake Forms (gender identity, sexual orientation, relationship status, parentage); Embryology - Gender versus Sex; Embryology - Changing Terminology; Gender dysphoria versus Transgender
Understanding typical (male and female) sex development and knowing the main etiologies of atypical sex development.	Embryology - Disorders of Sex Development (What are they? What are the current thoughts on treatment options? What are gender assignments?); Embryology - Gender versus Sex; Gender dysphoria versus Transgender
Understanding and explaining how stages of physical and identity development across the lifespan affect the above-described populations and how health care needs and clinical practice are affected by these processes.	Embryology - Disorders of Sex Development (What are they? What are the current thoughts on treatment options? What are gender assignments?); Embryology - Gender versus Sex
Apply principles of social-behavioral sciences to the provision of patient care, including assessment of the impact of psychosocial and cultural influences on health, disease, care seeking, care compliance, and barriers to and attitudes toward care by:	
Understanding and describing historical, political, institutional, and sociocultural factors that may underlie healthcare disparities experienced by the populations described above	Embryology - Disorders of Sex Development (What are they? What are the current thoughts on treatment options? What are gender assignments?); Embryology - Changing Terminology
Demonstrate an investigatory and analytic approach to clinical situations by:	
Recognizing the gaps in scientific knowledge (e.g., efficacy of various interventions for DSD in childhood; efficacy of various interventions for gender dysphoria in childhood) and identifying various harmful practices (e.g., historical practice of using “reparative” therapy to attempt to change sexual orientation; withholding hormone therapy from transgender individuals) that perpetuate the health disparities for patients in the populations described above.	Embryology - Disorders of Sex Development (What are they? What are the current thoughts on treatment options? What are gender assignments?); Embryology - Changing Terminology; Hormone Therapy Pharmacology; Transitioning options and associated risks; Puberty suppression in management of trans youth; Gender dysphoria versus Transgender
Competency Domain: Practice-Based Learning and Improvement	
Identify strengths, deficiencies, and limits in one’s knowledge and expertise by:	
Critically recognizing, assessing, and developing strategies to mitigate the inherent power imbalance between physician and patient or between physician and parent/guardian, and recognizing how this imbalance may negatively affect the clinical encounter and health care outcomes for the individuals described above.	Communication / Interview Skills; Standardized Patient Cases; Puberty suppression in management of trans youth
Demonstrating the ability to elicit feedback from the individuals described above about their experience in health care systems and with practitioners, and identifying opportunities to incorporate this feedback as a means to improve care (e.g., modification of intake forms, providing access to single-stall, gender-neutral bathrooms, etc.).	Intake Forms (gender identity, sexual orientation, relationship status, parentage); Standardized Patient Cases; Embryology - Disorders of Sex Development (What are they? What are the current thoughts on treatment options? What are gender assignments?); Embryology - Gender versus Sex; Embryology - Changing Terminology
Locate, appraise, and assimilate evidence from scientific studies related to patients’ health problems by:	
Identifying important clinical questions as they emerge in the context of caring for the individuals described above, and using technology to find evidence from scientific studies in the literature and/or existing clinical guidelines to inform clinical decision-making and improve health outcomes.	Communication / Interview Skills; Case-based-Learning integration; Exclusive WSWs: Pap smear, Breast Exams, and HPV Screening; Anal Pap smear; MSMs and need of Hepatitis A/HPV shot; Lesbian Obesity; Increased heart disease rate in lesbians; Anal Cancer Risks, Treatment, Anal Pap in MSM; Lesbian nulliparity and risk of breast/ovarian/cervical cancer; Eating disorders in MSM
Competency Domain: Interpersonal and Communication Skills	
Communicate effectively with patients, families, and the public, as appropriate, across a broad range of socioeconomic and cultural backgrounds by:	
Developing rapport with all individuals (patients, families, and/or members of the health care team) regardless of others’ gender identities, gender expressions, body types, sexual identities, or sexual orientations, to promote respectful and affirming interpersonal exchanges, including by staying current with evolving terminology.	Communication / Interview Skills; Standardized Patient Cases; Embryology - Changing Terminology
Recognizing and respecting the sensitivity of certain clinical information pertaining to the care of the patient populations described above, and involving the patient (or the guardian of a pediatric patient) in the decision of when and how to communicate such information to others.	Communication / Interview Skills; Puberty suppression in the management of trans youth
Demonstrate insight and understanding about emotions and human responses to emotions that allow one to develop and manage interpersonal interactions by:	
Understanding that implicit (i.e., automatic or unconscious) bias and assumptions about sexuality, gender, and sex anatomy may adversely affect verbal, nonverbal, and/or written communication strategies involved in patient care, and engaging in effective corrective self-reflection processes to mitigate those effects.	Assumptions/Biases Embryology - Disorders of Sex Development (What are they? What are the current thoughts on treatment options? What are gender assignments?); Embryology - Gender versus Sex; ID - STIs in lesbians (What do/can they get?); ID - Vaginitis spread in lesbians
Identifying communication patterns in the health care setting that may adversely affect the care of the described populations, and learning to effectively address those situations to protect patients from the harmful effects of implicit bias or acts of discrimination.	Assumptions/Biases; Standardized Patient Cases; Embryology - Gender versus Sex; Transitioning options and associated risks; Gender dysphoria versus Transgender
Competency Domain: Professionalism	
Demonstrate sensitivity and responsiveness to a diverse patient population, including but not limited to diversity in gender, age, culture, race, religion, disabilities, and sexual orientation by:	
Recognizing and sensitively addressing all patients’ and families’ healing traditions and beliefs, including health-related beliefs, and understanding how these might shape reactions to diverse forms of sexuality, sexual behavior, sexual orientation, gender identity, gender expression, and sex development.	Communication / Interview Skills
Demonstrate respect for patient privacy and autonomy by:	
Recognizing the unique aspects of confidentiality regarding gender, sex, and sexuality issues, especially for the patients described above, across the developmental spectrum, and employing appropriate consent and assent practices.	Embryology - Disorders of Sex Development (What are they? What are the current thoughts on treatment options? What are gender assignments?); Embryology - Gender versus Sex
Demonstrate accountability to patients, society, and the profession by:	
Accepting shared responsibility for eliminating disparities, and overt bias (e.g., discrimination), and developing policies and procedures that respect all patients’ rights to self-determination.	Assumptions/Biases; Substance Abuse Screening; ID - STI recommendations in MSM HIV in MSM; Lesbian Obesity; Increased heart disease rate in lesbians; Anal Cancer Risks, Treatment, Anal Pap in MSM; Lesbian nulliparity and risk of breast/ovarian/cervical cancer
Understanding and addressing the special challenges faced by health professionals who identify with one or more of the populations described above to advance a healthcare environment that promotes the use of policies that eliminate disparities (e.g., employee nondiscrimination policies, comprehensive domestic partner benefits, etc.).	Assumptions/Biases
Competency Domain: Systems-Based Practice	
Advocate for quality patient care and optimal patient care systems by:	
Explaining and demonstrating how to navigate the special legal and policy issues (e.g., insurance limitations, lack of partner benefits, visitation, and nondiscrimination policies, discrimination against children of same-sex parents, and school bullying policies) encountered by the populations described above.	Case-based-Learning integration; Gay couples and fertility options; LGBTQI patients and having children (medical options and legal concerns)
Coordinate patient care within the health care system relevant to one’s clinical specialty by:	
Identifying and appropriately using special resources available to support the health of the individuals described above (e.g., targeted smoking cessation programs, substance abuse treatment, and psychological support).	Gay teen issues (psychological/sexual/coming out/identity development/schooling); Depression and Suicide Rates in LGBTQI teens/adults; Eating disorders in MSM; LGBTQI Teen Issues
Identifying and partnering with community resources that provide support to the individuals described above (e.g., treatment centers, care providers, community activists, support groups, legal advocates) to help eliminate bias from health care and address community needs	Assumptions/Biases; ID - STIs in lesbians (What do/can they get?); ID - STI recommendations in MSM; HIV in MSM Availability/Efficacy of Rectal Microbicides; Gay teen issues (psychological/sexual/coming out/identity development/schooling); Depression and Suicide Rates in LGBTQI teens/adults; Eating disorders in MSM; Gay couples and fertility options LGBTQI patients and having children (medical options and legal concerns); LGBTQI Teen Issues
Participate in identifying system errors and implementing potential systems solutions by:	
Explaining how homophobia, transphobia, heterosexism, and sexism affect health care inequalities, costs, and outcomes.	Assumptions/Biases
Describing strategies that can be used to enact reform within existing health care institutions to improve care to the populations described above, such as forming an LGBTQI support network, revising outdated nondiscrimination and employee benefits policies, developing dedicated care teams to work with patients who were born with DSD, etc.	Embryology - Disorders of Sex Development (What are they? What are the current thoughts on treatment options? What are gender assignments?); Embryology - Gender versus Sex
Incorporate considerations of cost awareness and risk-benefit analysis in patient and/or population-based care by:	
Demonstrating the ability to perform an appropriate risk/benefit analysis for interventions where evidence-based practice is lacking, such as when assisting families with children born with some forms of DSD, families with prepubertal gender nonconforming children, or families with pubertal gender nonconforming adolescents.	Embryology - Disorders of Sex Development (What are they? What are the current thoughts on treatment options? What are gender assignments?); Hormone Therapy Pharmacology; Transitioning options and associated risks; Puberty suppression in management of trans youth; Gender dysphoria versus Transgender
Competency Domain: Interprofessional Collaboration	
Work with other health professionals to establish and maintain a climate of mutual respect, dignity, diversity, ethical integrity, and trust by:	
Valuing the importance of interprofessional communication and collaboration in providing culturally competent, patient-centered care to the individuals described above and participating effectively as a member of an interdisciplinary health care team.	Communication / Interview Skills; Case-based-Learning integration; ID - STIs in lesbians (What do/can they get?)
Competency Domain: Personal and Professional Development	
Practice flexibility and maturity in adjusting to change with the capacity to alter one’s behavior by:	
Critically recognizing, assessing, and developing strategies to mitigate one’s own implicit (i.e., automatic or unconscious) biases in providing care to the individuals described above and recognizing the contribution of bias to increased iatrogenic risk and health disparities.	Assumptions/Biases; ID - STIs in lesbians (What do/can they get?); ID - Vaginitis spread in lesbians

Step three: expert panel selection

A team of experts in the Salt Lake Valley region was invited via email to participate in the process. Acknowledging that there is no standardized training or accreditation specific to GSD healthcare delivery, we deemed our experts as leaders in curriculum design and/or GSD healthcare topics due to their work, professional experience, and research focus. This study was a regional and multi-disciplinary effort with experts recruited through convenience sampling methodology, facilitated by using a published directory and roster of healthcare providers registered with Outcare Health in our region. This international provider directory helps GSD individuals find GSD-affirming providers. Experts included healthcare providers specializing in or providing GSD-specific care, researchers in sexuality/gender health, and clinical psychologists specializing in GSD-specific care. Additionally, experts included all course directors and lecturers involved in the medical school curriculum as they were stakeholders in UME curriculum design and delivery at our institution. Medical student leaders in the LGBTQI and Allies student interest group and Free HIV Pre-exposure Prophylaxis Clinic were also invited, as they were stakeholders in the improved incorporation of GSD healthcare topics in UME curriculum design and represented the students’ insight for this study. Experts consented to be part of our study and were not incentivized to participate in the study. Experts never met or interacted directly to discuss this study. Responses were blinded during consolidation and organization processes. Although experts agreed to participate in our study, it was not compulsory for each expert to participate in each round of our consensus process. 

Step four: poll experts iteratively

VSOM learning items and AAMC competencies previously matched together in DeVita et al. [5], were used as our initial questionnaire for our Delphi process. We utilized the online Qualtrics survey tool for step four and five. In step four, round one, the expert panel was asked to review the published DeVita et al. [5] learning items with associated AAMC competencies [4] and provide suggestions, comment on missing content, or create their own learning objectives in relation to the initial questionnaire. Individual responses from each participating expert were requested and collected electronically via Qualtrics survey. The research team then organized and consolidated responses into learning objectives utilizing development strategies from the AMA [17]. In round two, the expert panel was asked to focus on the language of the learning objectives to ensure inclusivity and accuracy. The research team organized and consolidated these responses. In round three, experts individually rated each learning objective’s importance in UME using a 5-point Likert scale (1 = not at all important; 2 = slightly important; 3 = moderately important; 4 = very important; 5 = extremely important).

Step five: identify convergence of opinion

In the final round (step five, round four), experts were given the group’s mean and mode rating for each item, reminded of their own initial rating, and asked to make a final 5-point rating. Learning objectives rated 4 or 5 (“very important” or “extremely important”) by at least 80% of experts were determined to be at consensus (this criterion was determined a priori). Because of concern that there may still be too many objectives at 80% expert consensus, the research team decided to stratify further which objectives had 100% expert rating of either 4 or 5, thus achieving universal consensus. Our research project was reviewed and declared exempt by our institution’s IRB.

## Results

Expert panel selection

At the start of this study, 379 individuals were solicited through email. Although fifty-nine individuals agreed to participate in the study, thirty-one individuals engaged in at least one step of the iterative process. Each expert did not need to participate in each round of our study. Furthermore, experts could elect to participate in a later round without participating in an earlier round. 

Eighteen individuals participated in this first round, twenty individuals participated in this second round, twenty-five individuals participated in this third round, and fifteen individuals participated in the final round. Table 2 demonstrates the various educational degrees attained by our expert panel. 

**Table 2:** Educational degree characteristics attained by expert panel

Expert characteristics	Number
Physicians (Medical Degree and Doctor of Osteopathic Medicine)	33
Physician Assistants	5
Doctorate Degrees (including Ph.D., Doctorate of Pharmacy, Doctorate of Physical Therapy)	11
Master's Degree (including in Occupational Therapy and Social Work)	5
Medical Students (Medical Degree candidates)	5

Among the physician experts, two of our experts practice urology and plastic surgery, both focusing on gender-affirming surgery. Approximately 25 percent of health care provider experts (physicians and physician assistants) delivered GSD-specific health care needs (including medication management or surgical management of gender transition). Of the Doctorate of Philosophy experts, one expert attained a Doctorate of Pharmacy with experience working in gender-related medication management, one expert achieved a Master of Social Work focusing their work on helping GSD patients, and one expert attained a Doctor of Physical Therapy specializing in pelvic floor rehabilitation. Regarding medical educational roles, nearly half of the expert panel were involved in education delivery as a lecturer, pre-clinical course director, clinical course director, and dean of UME.

Achieving consensus

A core aspect of this project was structured development and iterative processing of information. The initial questionnaire was composed of a combination of the 30 AAMC competencies and overlapping 32 Vanderbilt learning objectives [4,5]. After round two, 79 learning objectives were created. The final consensus process eliminated 28 objectives, resulting in 51 learning objectives, as seen in Table 3. We organized learning objectives into problem-based categories, aligning them with clinical and basic science content.

**Table 3:** Final learning objectives List of learning objectives, organized by curricular topic, deemed important by most respondents in the expert panel.  Objectives marked with an asterisk are learning objectives rated as "very important" or "extremely important" by 100% of respondents. GSD: gender and sexually diverse, IUI: intrauterine insemination, IVF: in vitro fertilization, STI: sexually transmitted infections, HPV: human papilloma virus. DSD: differences in sexual development,

	Final Learning Objectives
	Terminology
1.	Define the following terms: gender expression, gender identity, gender discordance, gender nonconformity, gender dysphoria, transgender, intersex, sexual orientation, sexual identity, and sexual behavior.*
2.	Compare gender discordance, gender nonconformity, gender dysphoria, and transgender identity.*
3.	Communicate with gender and sexuality diverse individuals using language that is familiar and respectful to these individuals.*
	Barriers to Care
4.	Identify barriers to gynecologic health, including menstruation, that gender and sexually diverse (GSD) individuals with uteruses may experience.
5.	Identify barriers to reproductive care (such as IUI, IVF, surrogates, and adoption) that GSD individuals may experience.
6.	Identify insurance limitations and structural policies that negatively affect the healthcare of GSD individuals. (i.e. visitation and nondiscrimination policies, comprehensive domestic partner benefits, etc.)
7.	Describe the impact of psychosocial and cultural influences on health, disease, care-seeking, and care compliance by recognizing the barriers to and attitudes toward care. Describe the historical, political, institutional, and sociocultural factors that may underlie health care disparities experienced by GSD individuals.
8.	Identify how anti-GSD legislation can impact GSD child/teen development and wellbeing.
9.	Identify local and national resources for GSD individuals (such as Pride Center, Encircle House, Gender bands, etc.)
10.	Identify local suicide prevention resources for GSD individuals, including teens, in urban and rural settings.*
	Health Disparities
11.	Discuss the disparities in violence, aggression, and homicide rates of GSD persons with respect to the intersectionality of other minority identities.
12.	Explain the minority stress model in relation to gender and sexual orientation and its clinical associations.
	History Taking
13.	Outline the 7ps framework for sexual history taking by discussing partners, practices, past history of STIs, protection from STIs, HIV PrEP, pregnancy/family planning, plus/sexual satisfaction.*
14.	Describe when and where to ask for a GSD individual’s data, including an organ inventory. When is this relevant? When is it not relevant? (for example, is it relevant to perform an organ inventory for someone who stepped on a nail versus someone coming in for abdominal pain?).
15.	Conduct patient interviews and recommend healthcare screenings based on the presence of sexual organs.
16.	Describe how to perform substance abuse screenings.
17.	Collect detailed mental health information with an emphasis on suicide risk, especially in youth who identify as gender and sexuality diverse.
	Physical Exam
18.	Describe physical exam skills specific to individuals with vaginas, vulvas, or mammary glands such as vaginal pap smear, breast exams, and HPV screening.*
19.	Demonstrate trauma-informed physical exams for anal, pelvic, breast, and genital regions for gender and sexually diverse (GSD) individuals.*
20.	Describe physical exam skills specific to individuals with penises and understand how anatomy may vary with transgender and patients with differences in sexual development.*
	HIV, STIs, and Cancer
21.	Discuss indications, risks benefits, and management of HIV pre-exposure prophylaxis (PrEP).
22.	Discuss the need for PrEP across GSD populations.
23.	Identify risk factors for HIV transmission in gender and sexually diverse populations (i.e. anatomy, sexual practices, socioeconomic status, and history)
24.	Describe recommendations for hepatitis A/human papilloma virus (HPV) vaccination in GSD individuals.
25.	List common methods of prevention of sexually transmitted diseases (STDs) specific to gender and sexually diverse individuals.
26.	Understand STD screenings based on an individual patient's practices/partners/preferences.*
27.	Identify the reasons for triple site testing for chlamydia and gonorrhea.
28.	List the symptoms and differential diagnosis of pelvic pain and interventions.
29.	Identify appropriate cancer screening for patients on hormonal therapy (i.e. increased estrogen may place individuals at greater risk for breast cancer) and appropriate screening.
	Differences of Sexual Development and Mental Health
30.	Define differences in sex development and describe treatment options, including gender considerations.
31.	Explain unique issues that arise for a GSD teen (psychological/sexual/identity development/schooling).
32.	Identify key features of trauma-informed psychotherapy.
33.	Describe the disparities in mental health outcomes for GSD individuals.
34.	Explore the physical and verbal signs of inter-partner violence and abuse in GSD individuals.
	Transgender Healthcare
35.	Describe the requirements for gender-affirming healthcare with respect to diagnostic and identity labels, such as gender dysphoria, gender euphoria, transgender, and nonbinary.
36.	Explore how sex and gender may change during an individual's lifespan.
37.	Explain differences in sexual development (DSD) as they relate to intersex identity and gender identity.
38.	Identify current pharmacological regimens for hormone therapy, including options and associated risks.
39.	Describe how to start the discussion and what language to use with a patient who is transitioning genders.
	Cultural Humility
40.	Define and practice cultural humility and person-first language.
41.	Discuss social determinants of health with respect to the social support of and lifelong care for GSD individuals.
42.	Design quality improvement efforts to address inclusivity (e.g., modification of intake forms, providing access to single-stall, gender-neutral bathrooms, etc.)
43.	Identify outdated language and suggest newer language that promotes affirming care, equity, diversity, and inclusion.*
44.	Describe the impact of microaggressions, implicit bias, and assumptions on GSD patient care that have the potential to alienate GSD patients.
45.	Describe how physicians who create an environment where all patients feel welcome can better meet health care needs.*
	General GSD Concerns
46.	Identify and explain principles of privacy and confidentiality as they pertain to GSD adults, youth, and guardians of pediatric patients.
47.	Identify automatic and unconscious assumptions that are commonly made about GSD patients.
48.	Explain the history of structural oppression in homophobia, transphobia, heterosexism, and sexism.
49.	Explain how homophobia, transphobia, heteronormativity, and sexism affect healthcare inequalities, costs and outcomes.
50.	Describe the importance of diversity and representation in healthcare providers in positive patient outcomes.
51.	Describe the history of oppression in gender and sexuality-diverse populations in health care and how these practices perpetuate health disparities for GSD individuals.

In further evaluating the results, we found that the panel universally agreed that medical students must be proficient in particular objectives with 100% expert consensus. These objectives are delineated by an asterisk in Table 3. We then evaluated these eleven objectives and condensed them further into similar themed areas for utilization for curriculum. These are listed below in Table 4.

**Table 4:** Areas in which medical students must be proficient in before graduation. List of determined areas medical students must be proficient in before graduation.  This is determined by achieving 100% expert panel consensus. GSD: gender and sexually diverse.

	Areas in which medical students should be proficient:
1.	Describing GSD terminology broadly.
2.	Recognizing sexual practices and gender expression spectrums.
3.	Conducting an inclusive and individualized sexual history aligned with the patient’s partners, practices, goals, and risks.
4.	Conducting sensitive physical exams on gender-diverse individuals.
5.	Recognizing how sexually transmitted infection screenings aligned with sexual practices.
6.	Describing methods for suicide prevention and resources that support GSD individuals.
7.	Creating a welcoming environment for GSD individuals with the elimination of bias.

## Discussion

We conducted a regional Delphi study to develop a comprehensive list of learning objectives to teach medical students about GSD medical care. The 51 learning objectives were created using local expert consensus based on the 2014 AAMC implementing curricular and institutional climate changes to improve health care for individuals who are LGBTQI, gender nonconforming, or born with DSD competencies and matched learning items extracted from DeVita et al. [4,5]. Throughout this consensus process, experts agreed on the importance of differentiating between identity, attraction, and behavior of GSD individuals compared to identity alone. This resulted in the newly developed learning objectives identifying specific risks, anatomy, barriers to care, and health inequities unique to the GSD population. These learning objectives can potentially be used concurrently with other educational content. They can provide a GSD perspective into the UME curriculum that may have been omitted or overlooked.

We further identified eleven learning objectives that attained 100% consensus by our expert panel. Learning objectives that were agreed upon universally by our expert panel surround topics of gender identity, appropriate history-taking framework, physical exam utilization, and health risks associated with sexual activity with proper screening tools. These eleven identified objectives are most helpful as a first step to incorporating GSD topics into a UME curriculum that may be deficient. 

Topics that were eliminated by our panel included objectives that were too detailed and granular for the UME curriculum. Topics that attained at least 80% consensus but not 100% universal voting included objectives grouped within healthcare disparities, differences in sexual development and mental health, transgender healthcare, and general GSD issues. Again, we speculate whether some of our expert panel members viewed these objectives as too detailed for UME learners and thus did not achieve unanimity. These objectives may have been rated differently by an expert panel in another geographical region with varied cultural and political dynamics. 

Interestingly, we did note that many learning objectives specifically related to transgender care were either eliminated or did not achieve 100 percent consensus with our expert panel. It is unclear why gender-transitioning options, such as hormonal medication utilization or gender-affirming surgeries, were not selected in consensus. This may point to the need to perform a separate research investigation evaluating UME objectives specifically addressing transgender health care. 

Much of the literature surrounding improving GSD medical education focuses on institution-specific interventions like pathway development or curricular modification. Few publications report a comprehensive approach to GSD health care designed for UME. Comprehensive UME resources that do exist focus primarily on assessment and integration process models or only on transgender/gender non-conforming healthcare [14,18-20]. While our learning objectives overlap with the 30 AAMC competencies, they are more concise and organized explicitly for immediate curriculum integration. Our study provides a clear set of GSD health objectives written in an actionable and easily accessible format compared to what currently exists in the literature. This actionable format exists because of our Delphi process with consensus from our experts. 

Limitations

We recognize there are limitations to our study. Our content may be limited since experts are from a single region. Additionally, we had a low response rate, with less than 10% of individuals participating in the study of the 379 individuals initially invited. Not all 31 individuals who participated in the study participated in every Delphi process round and experts could participate in a later round without participation in earlier rounds. We acknowledge that because of this, non-response bias could have skewed our final objectives. 

## Conclusions

Our study presents a newly designed, accessible format of GSD UME content ready for immediate utilization. Our learning objectives will aid in standardizing GSD health education in UME. We hope that utilizing these learning objectives will improve the knowledge of future health professionals and lead to improved health equity for GSD persons. Our next steps include developing auditing guidelines for these learning objectives to ensure medical students are trained to care for GSD individuals competently.
